# First line chemotherapy with planned sequential administration of gemcitabine followed by docetaxel in elderly advanced non-small-cell lung cancer patients: a multicenter phase II study

**DOI:** 10.1038/sj.bjc.6604187

**Published:** 2008-01-22

**Authors:** C Tibaldi, E Vasile, A Antonuzzo, R Di Marsico, A Fabbri, F Innocenti, G Tartarelli, D Amoroso, M Andreuccetti, M Lo Dico, A Falcone

**Affiliations:** 1 Division of Oncology, Department of Oncology, UO Oncologia Medica, Presidio Ospedaliero, Azienda USL-6 of Livorno Viale Alfieri 36, Livorno 57100, Italy; 2 Department of Oncology Civil Hospital, Via Forlanini 22, Piombino 57025, Italy; 3 Department of Oncology Civil Hospital, Via O. Marrucci 66, Cecina 57023, Italy; 4 Division of Pneumology Civil Hospital, Viale Matteotti 35, Pistoia 51100, Italy; 5 Division of Oncology, Department of Oncology Civil Hospital, Via Aurelia 335, Viareggio 55041, Italy; 6 Department of Oncology, University of Pisa Via Roma 67, Pisa 56100, Italy; 7 Istituto Toscano Tumori (ITT) Via Taddeo Alderotti 26/N, Firenze 50139, Italy

**Keywords:** gemcitabine, docetaxel, advanced NSCLC, elderly patients

## Abstract

This multicenter phase II study evaluated, in chemonaive patients with stage IIIB–IV NSCLC, age ⩾70 and with a performance status 0–2, the activity, efficacy and tolerability of planned sequential administration of gemcitabine 1200 mg m^−2^ on days 1 and 8 every 3 weeks for three courses followed by three cycles of docetaxel 37.5 mg m^−2^ on days 1 and 8 every 3 weeks, provided there was no evidence of disease progression. A total of 56 patients entered the study. According to intention-to-treat analysis, the objective response rate was 16.0% (95% CI 7.6–28.3%); 23 patients (41.0%) had stable disease and 24 patients (43%) had progressive disease. Five patients who had a stable disease after three courses of gemcitabine obtained a conversion to partial response by docetaxel. Median time to progression was 4.8 months (95% CI 3.6–6.0 months) and median duration of survival was 8.0 months (95% CI 5.6–10.5 months). The 1-year survival rate was 34%. No grade 4 haematological toxicity was observed and grade 3 neutropenia and thrombocytopenia were reported in 5.4 and 3.6% of the patients, respectively. Grade 3/4 mucositis and grade 3 diarrhoea, both occurred in 3.6% of the patients and grade 3 asthenia was observed in 9% of patients. One patient reported a grade 4 skin toxicity. No treatment-related deaths occurred. Sequential gemcitabine and docetaxel is a well-tolerated and effective regimen in elderly advanced NSCLC patients.

Lung cancer is the leading cause of cancer-related deaths in Western countries. Non-small-cell lung cancer, (NSCLC) accounts for 87% of primary lung cancers and approximately two-thirds of NSCLC patients are in an advanced stage at diagnosis (Govindan *et al*, 2006). Most malignancies, including lung cancer, occur more commonly in the elderly, with almost 50% diagnosed in patients aged ⩾70 years (Havlik *et al*, 1994).

Although third-generation doublets, platinum-based regimens, represent the gold standard in the treatment of advanced NSCLC patients who have good performance status (PS), most elderly advanced patients may be more susceptible to the toxic side effects of platinum-containing combinations due to higher rates of comorbid illness and the age-related impairment of organ function so that single-agent chemotherapy, such as gemcitabine, is considered a valid therapeutic choice (Gridelli *et al*, 2003; Pfister *et al*, 2004). Moreover, the large randomised phase III MILES-trial concluded that the non-platinum containing regimen vinorelbine plus gemcitabine did not provide a survival benefit over single-agent vinorelbine or gemcitabine, and that the two-drugs combination was more toxic than single-agent therapy (Gridelli *et al*, 2003).

Among the last generation drugs tested in NSCLC, docetaxel seems very promising. This drug has shown single-agent efficacy as a second-line treatment: in patients pre-treated with platinum-based chemotherapy as a first-line therapy, single-agent docetaxel proved to have a survival advantage over best supportive care alone (Shepherd *et al*, 2000), and in comparison to vinorelbine or ifosfamide alone (Fossella *et al*, 2000).

This drug also showed activity as a single agent in untreated elderly advanced NSCLC patients enrolled in the West Japan Thoracic Oncology Group Phase III Trial (WJTOG 9904), comparing docetaxel 60 mg m^−2^ to vinorelbine 25 mg m^−2^ on days 1 and 8 every 3 weeks. Indeed a statistically significant advantage in terms of progression-free survival (5.5 *vs* 3.1 months), response rate (22.7 *vs* 9.9%), and improvement of disease-related symptoms was observed in favour of docetaxel; docetaxel had a non-significantly prolonged median overall survival (14.4 *vs* 9.9 months) (Kudoh *et al*, 2006). In the first line setting, docetaxel demonstrated both an interesting activity and efficacy whether combined with a platinum compound (Fossella *et al*, 2003) or with gemcitabine. In particular, among non-platinum-based chemotherapy regimens, the combination docetaxel–gemcitabine is considered one of the most promising. In fact, a randomised phase II trial and two randomised phase III trials compared this association with last generation platinum-containing doublets, docetaxel–cisplatin (Georgoulias *et al*, 2001) and cisplatin–vinorelbine (Georgoulias *et al*, 2005; Pujol *et al*, 2005) yielding similar activity and efficacy. Therefore, the combination docetaxel–gemcitabine could be considered a regimen to test in elderly subjects, as tolerability, especially haematological toxicity, can be problematic (Georgoulias *et al*, 2005; Pujol *et al*, 2005; Manegold *et al*, 2006).

A possible approach to reduce the toxicity of combination regimens consists of administering the same drugs in a sequential manner. This strategy may allow the administration of full dose single agents sequentially without compromising efficacy, while reducing potential toxicity expected with concurrent administration. Preclinical models (Norton and Simon, 1977; Day, 1986) as well as recent clinical trial in NSCLC suggest a benefit for the sequential administration of chemotherapy agents. A randomised phase III trial on advanced NSCLC (Manegold *et al*, 2006), having clinically relevant haematological toxicity (defined as thrombocytopenia with platelets transfusions, anaemia with RBC-transfusions or febrile neutropenia) as a primary end point, compared a concomitant administration of gemcitabine 1000 mg m^−2^ on days 1 and 8 and docetaxel 75 mg m^−2^ on day 1 every three weeks for 6 courses *vs* their sequential administration (3 courses of gemcitabine followed by 3 courses of docetaxel at the same dosages). Clinically relevant haematological toxicity occurred less frequently in the sequential arm and the quality of life also improved with this approach.

Three randomised phase III trials (Gridelli *et al*, 2004; Schuette *et al*, 2005; Camps *et al*, 2006) have compared, in second line advanced NSCLC, docetaxel at the dose of 75 mg m^−2^ every 3 weeks, considered the standard of care, against a weekly schedule, indicating a similar efficacy but a significantly less severe toxicity in terms of leukopenia, neutropenia and febrile neutropenia with weekly docetaxel.

We carried out a multicenter phase II study to evaluate the activity, efficacy and tolerability, of a sequential regimen consisting of three courses of gemcitabine followed by three courses of docetaxel provided there was no evidence of disease progression. The schedule chosen for docetaxel was 37.5 mg m^−2^ on days 1 and 8 every 3 weeks according to our previous study carried out in elderly patients in a second-line setting (Tibaldi *et al*, 2006; Gridelli *et al*, 2007b).

## PATIENTS AND METHODS

### Patient selection criteria

Chemotherapy-naive patients with histologically or cytologically confirmed NSCLC, aged ⩾70 years, measurable disease, and clinical stage IIIB (cytologically positive pleural effusion or metastatic supraclavicular lymph nodes) or stage IV disease were eligible if they also met the following criteria: Eastern Cooperative Oncology Group PS ⩽2, life expectancy >3 months, adequate bone marrow reserve (leukocyte count ⩾4.0 × 10^9^ l^−1^, platelet count ⩾100 × 10^9^ l^−1^), adequate hepatic function (bilirubin level ⩽1.5 mg dl^−1^) and renal function (creatinine level ⩽1.5 mg dl^−1^). Prior radiotherapy was allowed provided that the irradiated area was not the only source of measurable disease and that radiation therapy had been completed 7 days before chemotherapy was initiated.

Patients were excluded for the presence of active infections, concomitant malignancy, or a second primary malignancy, recent myocardial infarction, unstable angina, congestive heart failure, dementia, symptomatic brain metastases. A written informed consent was obtained from each patient before enrolment. The protocol was approved by each local ethics committee of every institution participating to the study and the trial was conducted according to the Helsinki declaration of the World Medical Association.

### Treatment

The chemotherapy regimen consisted of gemcitabine (Gemzar®, Eli Lilly and Company, Indianapolis, Indiana, USA) 1200 mg m^−2^ in 250 ml of normal saline administered intravenously over 30 min on days 1 and 8 every 3 weeks for 3 courses followed by, docetaxel (Taxotere®, Aventis Pharma, Antony Cedex, France) 37.5 mg m^−2^ in 500 ml of normal saline administered intravenously over 60 min on days 1 and 8 every 3 weeks for 3 courses, provided there was no evidence of disease progression; premedication consisted of 20 mg dexamethasone i.v. and 5-hydroxytryptamine-3 receptor antagonists as antiemetic prophylaxis. Patients whose white blood cell count, neutrophil count and platelet count were greater than 3.5 × 10^9^, 1.5 × 10^9^ and 100 × 10^9^ l^−1^, respectively, received chemotherapy on day 1. If these conditions were not met, administration of chemotherapy was delayed 1 week or until recovery. A dose delay for more than 3 weeks resulted in withdrawal from the study. The dose of chemotherapy (gemcitabine and docetaxel) was modified on day 8 according to haematological and non-haematological toxicities as follows: if neutrophil count was >1.5 × 10^9^ l^−1^ and platelet count was >100 × 10^9^ l^−1^, chemotherapy was administered at full dose; for neutrophil count 1.0–1.49 × 10^9^ l^−1^ or platelet count 75–99 × 10^9^ l^−1^, the dose was reduced by 25%; for neutrophil count 0.5–0.99 × 10^9^ l^−1^ or platelet count 50–74 × 10^9^ l^−1^ the dose was reduced by 50%; for neutrophil count <0.5 × 10^9^ l^−1^ or platelet count <50 × 10^9^ l^−1^, chemotherapy was omitted. If grade 2 non-haematological toxicity (except for alopecia) was observed, chemotherapy was omitted until resolution and then readministered at the next cycle at doses reduced by 25–50%. In case of grade 2 neurological toxicity or grade 3–4 non-haematological toxicity the patient was withdrawn from the study.

The routine use of granulocyte colony-stimulating factor was not allowed.

### Evaluation criteria

Pretreatment evaluation included medical history, physical examination, assessment of PS, complete blood cell count with differential, routine chemistry, chest radiograph, computed tomography (CT) scan of the chest and abdomen. A comorbidities assessment using the Charlson comorbidity index was carried out (Charlson *et al*, 1987). In particular, the presence or absence of the following disease states was recorded: myocardial infarction, peripheral vascular disease, cerebrovascular disease, chronic pulmonary disease, connective tissue disease, ulcer disease, mild liver disease, diabetes, hemiplegia; each disease state had a corresponding numeric value (Charlson *et al*, 1987), and the value for all present disease states were summed for each patient. The other pathologic conditions (congestive heart failure, dementia, moderate or severe kidney disease, diabetes with organ damage, any other cancer, moderate or severe liver disease, AIDS), used to calculate the Charlson score, were not taken into account because they were precluded by the exclusion criteria for the study.

During treatment, a complete blood cell count was performed before each chemotherapy administration. Eastern Cooperative Oncology Group PS was evaluated at each cycle. Evaluation of tumour response was carried out with CT scan every three cycles. Responses were assessed using standard RECIST criteria (Therasse *et al*, 2000). The best overall response for each patient was reported; all results were reviewed by an independent radiologist and had to be confirmed 28 days or more after initial documentation of the response. Patients with disease progression after 3 courses of gemcitabine were considered as such in the final analysis. The overall response rate was calculated according to the intention-to-treat analysis.

Haematological toxicity and non-haematological toxicities were recorded at days 1 and 8 of every course of treatment. The worst toxicity grade for each patient in all cycles was reported. Toxicities were assessed using National Cancer Institute common toxicity criteria version 2.0 (NCI-CTC) (National Cancer Institute, 1999).

Quality of life was assessed using the European Organisation for Research and Treatment (EORTC) QLQ-C30 questionnaire (version 3.0) and the lung cancer-specific module (QLQ-LC13). Patients were asked to complete the questionnaires at baseline and at the end of every cycle.

### Statistical analysis

The main objective of the study was to test whether the sequential combination of gemcitabine and docetaxel would improve response rate. Secondary end points were to evaluate toxicity, time to progression and survival. Simon's two-stage minimax design was applied to calculate the sample size. Assuming p_0_ (low response rate) 20% and *p*_1_ (target response rate of interest for further investigation) 35% and with an *α* error of 0.05 and a *β* error of 0.20 a total of 31 evaluable patients had to be accrued during stage 1. If at least six objective responses were observed, 22 additional patients were to be enrolled into the study during stage 2. The regimen would be considered for further investigation if ⩾15 objective responses out of 53 evaluable patients were observed.

Time to progression (TTP) was calculated from the date of registration to the date of clinical and/or radiological evidence of progression or death, whichever occurred first. Survival was calculated from registration to death or last follow-up. Survival and TTP were estimated using the Kaplan–Meier method.

Since most patients had more than one comorbid medical condition, the comorbidities count was dichotomised as 0–1 or >1 whereas Charlson score was dichotomised as 0 or ⩾1, as reported previously (Hesketh *et al*, 2006), for survival comparisons. Cox regression analyses were used to examine any possible relationships between overall survival and comorbidities count or Charlson score.

Data were analysed using SPSS/PC+11.5statistical software (SPSS Inc., Chicago, IL, USA).

## RESULTS

### Patient characteristics

From March 2005 to October 2006, 56 patients entered the study (Table 1). Forty-six patients (82%) were males, 10 (18%) were females; median age was 76 years (range 70–84). Seven patients (12.5%) had a PS=0, 38 patients had a PS=1 (67.8%), 11 patients had a PS=2 (19.6%). Squamous carcinoma was the most frequent histology (20 patients); 17 patients had adenocarcinoma, one patient had bronchioloalveolar carcinoma, four patients presented large cell carcinoma and 14 patients had undifferentiated NSCLC. All patients had stage IV disease. Table 2 shows the distribution of comorbid conditions.

### Dose administration

Fifty-six patients received a total of 243 cycles, 159 cycles of gemcitabine and 84 cycles of docetaxel; the median number of total courses (gemcitabine plus docetaxel) was 5 (range, 1–6). Thirty-two patients received docetaxel and the median number of docetaxel courses was 3 (range 1–3). The delivered dose-intensity for gemcitabine and docetaxel was 690.4 and 20.3 mg m^−2^ per week, respectively, whereas the relative dose intensity was 86.2 and 81.2%, respectively. Twelve delays (7.5% of the courses) were reported during gemcitabine treatment. Toxicity was the reason for the delays in only three cases (1 episode of grade 2 neutropenia, 1 episode of grade 2 skin toxicity and 1 episode of AST and ALT elevation); the reasons for the other nine delays were fever in three cases and scheduling conflict in six cases. Seventeen delays (20.2% of the courses) were reported during docetaxel treatment. The reasons for the delays were one episode of grade 3 mucositis, two episodes of grade 3 diarrhoea, one episode of grade 3 neutropenia, two episodes of grade 2 skin toxicity, one episode of creatinine increase and one episode of bilirubin increase; in nine cases the reasons were not related to toxicity (fever in five cases and scheduling conflict in four cases). Six patients discontinued treatment due to docetaxel toxicity: one patient experienced grade 4 mucositis, one patient had grade 3 mucositis, one patient experienced grade 4 skin toxicity, two patients reported grade 3 asthenia and one patient had an allergic reaction; three patients didn't discontinue treatment although they reported grade 3 asthenia. The gemcitabine dose was reduced by 25% in six administrations whereas the docetaxel dose was reduced by 25% in 16 administrations, and by 50% in four administrations.

### Response and survival

Fifty-three out of 56 patients were evaluable for response. Three patients could not be evaluated for the following reasons: one patient refused to continue chemotherapy after the first cycle, one patient died before the first evaluation probably due to rapid tumour progression, one patient was lost to the follow-up; these three patients were considered as progression disease in the final analysis.

According to the intention-to-treat analysis, the overall response rate was 16.0% (9 out of 56 patients) (95% CI 7.6–28.3%); twenty-three patients (41.0%) had stable disease and twenty-four patients (43%) had progressive disease. Five patients who had a stable disease after three courses of gemcitabine obtained a conversion to partial response by docetaxel.

**Figure 1:**
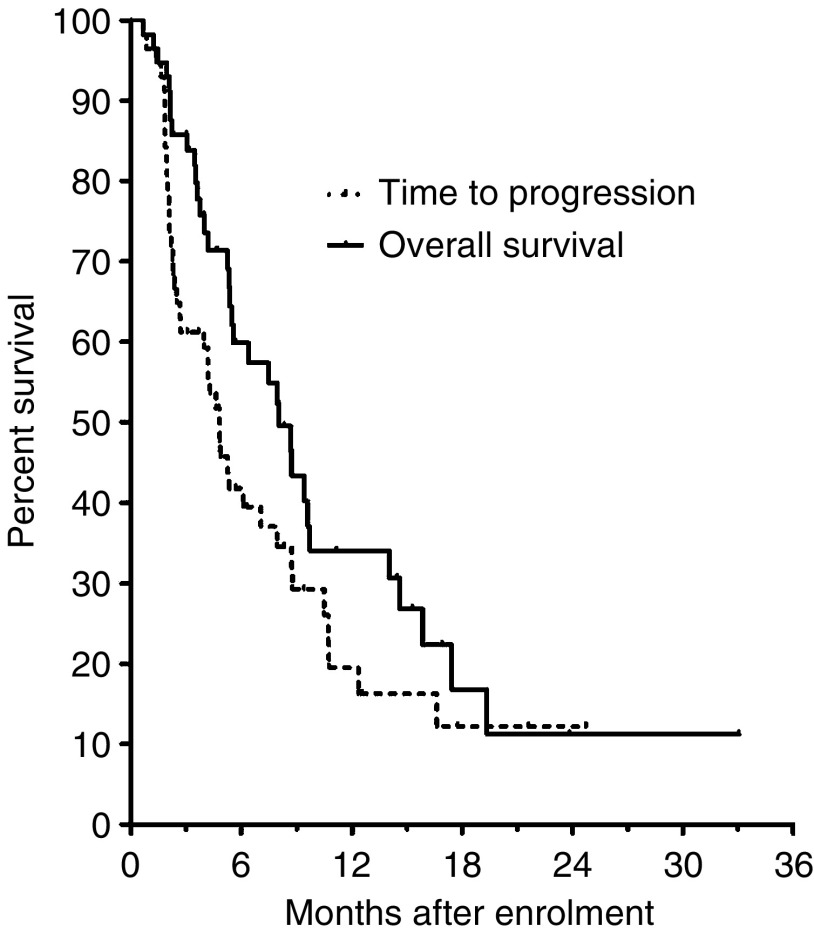
Kaplan–Meier plot of overall survival (OS) and time to progression (TTP).

Median time to progression was 4.8 months (95% CI 3.6–6.0 months) and median duration of survival was 8.0 months (95% CI 5.6–10.5 months). The 1-year survival rate was 34% (Figure 1). At an exploratory planned analysis, the subgroup of patients with PS 0–1 had a median time to progression of 4.8 months (95% CI 2.6–7.0 months), a median duration of survival of 8.7 months (95% CI 7.4–9.9 months) and 1-year survival rate of 40%; patients with PS 2 reported a median time to progression and a median overall survival of 4.0 months (95% CI 0.6–7.3 months) and 5.4 months (1.3–9.4 months), respectively, with a 1-year survival rate of 15%.

Patients with a Charlson score of 0 (18 patients) or ⩾1 (38 patients) had a median survival of 9.7 months and 7.9 months, respectively (*P*=0.82).

Since several comorbidities were not taken into account by Charlson score we analysed the median survival according to the number of comorbidities: the median survival was 8.6 months for patients with 0–1 comorbidities and 5.4 months for patients with >1 comorbidities (*P*=0.64).

Although planned, quality of life assessment isn't reported because of the lacking of sufficient data due to the high rate of missing questionnaires.

### Toxicity

All 56 patients were evaluable for toxicity. Observed toxicities were mild and the compliance to treatment was good. No grade 4 haematological toxicity was observed and grade 3 neutropenia and thrombocytopenia were reported in 5.4 and 3.6% of the patients, respectively. No patients developed febrile neutropenia or haemorrhages. Non-haematological toxicity consisted mainly in grade 3/4 mucositis and grade 3 diarrhoea both occurring in two (3.6%) patients, grade 3 asthenia observed in five (9%) patients. One patient reported a grade 4 skin toxicity and two patients reported a grade 3 nail toxicity. No treatment-related deaths occurred. Haematological and non-haematological toxicities are summarised in Table 3.

## DISCUSSION

The present trial, targeting elderly (age 70 years and older) advanced-stage NSCLC patients, was designed to test the hypothesis that planned sequential administration of gemcitabine followed by docetaxel may improve response rate with good tolerability. We hypothesised that the introduction of docetaxel in our first line sequential regimen could enhance the response rate with respect to our previous trial carried out in 110 elderly advanced NSCLC patients treated with gemcitabine alone (Tibaldi *et al*, 2005). In the present trial, the overall response rate according to an intention to treat analysis was 16.0% that was similar to our previous trial (13.9%). Nevertheless, it is remarkable that five out of nine responses that we observed were obtained from a conversion of stable disease to partial response by docetaxel.

We observed a median time to progression (TTP) of 4.8 months, a median overall survival (OS) of 8.0 months, and 1-year survival rate of 34%, that can be considered encouraging. In our previous trial, in fact, we reported a median TTP of 3.2 months, a median OS of 5.4 months and 1-year survival rate of 27% (Tibaldi *et al*, 2005). In addition, the percentage of PS 2 patients enrolled in both trials was similar at 20%.

The introduction of docetaxel in first-line treatment of advanced NSCLC resulted advantageous in progression-free survival in a recent randomised phase III trial that compared immediate docetaxel with docetaxel upon evidence of progression in non-progressing patients after four cycles of induction therapy with gemcitabine plus carboplatin. The progression-free survival in the immediate docetaxel arm (6.5 months) was significantly greater (*P*=0.0001) than in the delayed docetaxel arm (2.8 months) (Fidias *et al*, 2007).

Haematological and non-haematological toxicities, in our study, were mild and acceptable. The compliance to treatment was in general good. In particular, the present trial confirms our previous observation (Tibaldi *et al*, 2006) that a modified schedule of docetaxel (37.5 mg m^−2^ on days 1 and 8 every 3 weeks) is feasible and well tolerated in elderly advanced NSCLC patients. Our tolerability data confirm the previous observations that weekly docetaxel is advantageous in terms of haematological toxicity with respect to 3-weekly schedule (Gridelli *et al*, 2004; Schuette *et al*, 2005; Camps *et al*, 2006).

Recently, the results of the SWOG (S0027) phase II trial using a sequential regimen of vinorelbine 25 mg m^−2^ on days 1 and 8 every 3 weeks for three cycles followed by three courses of docetaxel 35 mg m^−2^ on days 1, 8, 15 every 4 weeks in patients 70 years of age and older and in patients with PS two of any age were published (Hesketh *et al*, 2006). Patients aged ⩾70 years with a PS of 0–1 had a median TTP and a median OS, of 4.7 and 9.1 months respectively, comparable to our results. However, some toxicities were more common such as grade 3/4 neutropenia and grade 3/4 fatigue, seen in 32 and 22% of patients, respectively. Another recent randomised phase II trial evaluated chemotherapy with pemetrexed alone *vs* a sequential pemetrexed/gemcitabine regimen in patients who were elderly (⩾70 years) or younger than 70 years and ineligible for platinum-based chemotherapy. The median TTP and OS reported were lower than expected and equal, in both arms, to 4.1 and 5.4 months, respectively (Gridelli *et al*, 2007a). A possible explanation for these results might be the high proportion of PS 2 patients (35.6%), who have poorer prognosis, enrolled into this study. However, although some trials grouped elderly patients with PS 2 patients of any age, these cohorts of patients have probably different characteristics and prognosis and should be included in different and specific dedicated clinical trials in the future.

Two multicenter phase II trials evaluated a weekly combination regimen of docetaxel and gemcitabine in advanced NSCLC; the first trial (Neubauer *et al*, 2005) reported a response rate of 20%, a median TTP and OS of 5.1 and 6.9 months, respectively, that favourably compare with our results; the second trial, that used a patient selection criteria based on age, PS and Charlson score, reported a higher response rate of 34%, but a similar median TTP and OS of 5 and 7 months, respectively (LeCaer *et al*, 2007). However, the frequency of grade III–IV fatigue was high and reported in 30% of patients.

In our study, we did not observe significant differences in overall survival according to Charlson score (0 or >1); although, the Charlson score did not correlate with PS (Dujon *et al*, 2006), it seems to be insufficient to screen elderly advanced NSCLC patients (Maione *et al*, 2005). The use of a comprehensive geriatric assessment, according to the recommendations of the SIOG (Extermann *et al*, 2005), appears crucial to improve the selection and stratification of elderly patients and thereby to allow valid comparisons among different studies.

In this setting, quality of life evaluation has prognostic value for survival (Maione *et al*, 2005); nevertheless, in our study we did not report this analysis because of a lack of sufficient questionnaires and quality of life assessment should be recommended in future trials.

At present, the role of platinum-based combinations in elderly advanced NSCLC is not clear; retrospective subset analyses of a number of phase III trials suggested that combination platinum-based therapy is superior to single agents in both younger and older patients (Langer *et al*, 2002; Lilenbaum *et al*, 2005); however, prospective randomised trials are lacking to confirm this hypothesis and the results of the MILES-2 study, comparing gemcitabine *vs* cisplatin/vinorelbine and *vs* cisplatin/gemcitabine, are eagerly awaited.

In conclusion, our results suggest that the sequential use of gemcitabine and docetaxel is another viable, option for elderly advanced NSCLC patients. The use of standardised specific geriatric evaluations appears to be crucial for future trials in this setting.

## Figures and Tables

**Table 1:** Clinical characteristics

	**No of patients (%)**
No of Patients:	56
Age, years (range)	76 (70–84)
	
*Sex*	
Male	46 (82)
Female	10 (18)
	
*Smoking history*	
Smokers	48 (85.7)
Never smokers	8 (14.3)
	
*ECOG PS*	
0	7 (12.5)
1	38 (67.8)
2	11 (19.6)
	
*Histology*	
Adenocarcinoma	17 (30.4)
Squamous	20 (35.7)
Large cell	4 (7.1)
Bronchioloalveolar	1 (1.8)
Unspecified NSCLC	14 (25.0)
	
*Metastatic sites*	
Bone	14 (25.0)
Brain	6 (10.7)
Liver	4 (7.1)
Adrenal gland	7 (12.5)
Lymphonodes	29 (51.8)
Pleura	11 (19.6)
Lung	14 (25.0)
	
*Number of comorbidities*	
None	3 (5.3)
1	21 (37.6)
2	15 (26.7)
3	12 (21.4)
4	5 (9)
	
*Charlson score*	
0	18 (32.1)
1	28 (50.0)
2	10 (17.8)

**Table 2:** Comorbid conditions

	**No of patients (%)**
Myocardial infarction	5 (8.9)
Ischaemic heart disease	6 (10.7)
Valvular heart disease	1 (1.8)
Atrial fibrillation	5 (8.9)
Peripheral vascular disease	3 (5.4)
Cerebrovascular disease	6 (10.7)
Hypertension	26 (46.4)
Chronic pulmonary disease	11 (19.6)
Mild liver disease	3 (5.4)
Peptic ulcer	7 (12.5)
Diabetes	12 (21.4)
Connective tissue disease	1 (1.8)
Osteoporosis	5 (8.9)
Mild depression	2 (3.6)
Mild kidney disease	4 (7.1)
Genitourinary diseases	10 (17.9)

**Table 3:** Haematological and non-haematological toxicity per patient

**NCI-CTC grade % (56 patients)**				
	**1**	**2**	**3**	**4**
Neutropenia	12.5	10.7	5.4	0
Thrombocytopenia	8.9	1.8	3.6	0
Anaemia	46.4	18	0	0
Nausea/vomiting	23.2	3.6	0	0
Diarrhoea	14.3	3.6	3.6	0
Mucositis	16.1	3.6	1.8	1.8
Asthenia	20	32	9	0
Nail toxicity	15	3.6	–	–
Skin toxicity	5.4	3.6	0	1.8
